# Annotations

**Published:** 1897-07-31

**Authors:** 


### Annotations.

**Medical Officers and their Reports.**

The Vestry of St. George has tried to spite its medical officer, and has succeeded in making itself ridiculous. However, it has been called to order by the Local Government Board and threatened with divers penalties, so we must hope it will perform its statutory duties more satisfactorily in future. The particular means of annoying Dr. Waldo which the vestry adopted in this instance was a peculiarly inept one, and consisted in refusing to print his report until it had been referred to the Health Committee, as if a report edited and curtailed by such a committee would be accepted by the Local Government Board or would be of the slightest value to anyone. The question of the freedom of the medical officer to say what he likes in his report is a matter of interest quite outside and beyond the petty politics of Southwark. The medical officer of health occupies the position of an adviser rather than a mere servant to a sanitary authority, and his report is a public document giving information in regard to the sanitary condition of the district to which both the ratepayers and the Local Government Board have a statutory right of access. It is possible that medical officers of health have sometimes padded their reports with matters of but small novelty although of considerable educational value. That, however, is an ephemeral affair, a mere incident in the progress of sanitation, and is sure to cease as sanitary authorities increase in knowledge of sanitary affairs. But so far as a complete presentment of the exact condition of the districts under their charge is concerned nothing could be more foolish than any attempt at docking the medical officer's report. Few things can be more useful in guiding the action of a sanitary authority than the continuous record and the proper arrangement of the vital and other statistics which come under the ken of a careful medical officer of health. Of this we have a good example in the admirable reports issued year by year by the medical officer to the London County Council. But even from the very meanest of motives, and from a mere desire to be assured as to the work that he has done, a sanitary authority should always encourage their medical officer to send in a complete and full report. As to the printing of it, it is ordered by the Public Health Act, 1891, so far as London is concerned, that the report of the medical officer of health must be affixed to that of the sanitary authority itself; the latter, in fact, is incomplete without the former.

**Death Rates and Health.**

The Chairman of the London County Council says that for the decade 1885-94 the death-rate of London has been lower than that of any of the capitals of Europe. At his request the medical officer of health has made a calculation which he thinks is interesting. He has compared two periods of seven years, one before and one since the Local Government Act of 1888, viz., 1882-88 with 1889-95. The result shows that had the rate of mortality observed in the earlier period been maintained in the later one, there would have been 13,294 more deaths in the later period than actually occurred. This saving of life has been mostly at the younger ages, and, he says, seems to show that the sanitary efforts made in recent years by the local sanitary authorities in co-operation with the Council have not been unfruitful. So far as it goes, as a bald statement of fact, this is very satisfactory. We would, however, warn our readers not to attribute too much importance to these crude statistical statements. With a lessening birth-rate and a growing population, along with a constant influx from the country—in other words, with a population whose losses from death are partly replaced by young adults instead of by babies, a lowering of the death-rate may well occur from a change in the relative importance of these factors, wholly irrespective of any improvement in health conditions.

**The Enrichment of Gas.**

It is well known that the "gas" supplied by the gas companies is much sophisticated, being often diluted to a large extent with water-gas, a substance which is very poisonous, very cheap, and, from an illuminating point of view, very poor. When the gas companies came into existence people did not think of these things. "Gas" was thought to be the product of the destructive distillation of coal, refined no doubt, but with nothing added, and the Acts by which the companies are regulated merely provide on the one hand that it shall possess a certain illuminating power, and on the other that the amount of impurities that it contains shall not exceed a certain limit. Hence the present condition of affairs. If gas were used only for outdoor illumination it would not matter, but for the past fifty years the untutored British plumber has been engaged in stuffing our dwelling-houses with leaky gas-pipes, and the poisonous nature of a great deal of our modern gas is a serious matter. It acts directly on the blood corpuscles, and in such a way that it is impossible to say where the mischief ends which is caused by the constant breathing of a gas-laden atmosphere. To add insult to injury, we now find that this poisonous gas cheats our pockets as well as injuring our persons. As is well known, the gas supplied to the metropolis is periodically tested at certain "testing stations," and if the gas were gas, as it used to be, it would no doubt arrive at the house of the consumer in the same condition as when it was passed by the gas examiner. But then gas is not gas. Its illuminating power to a large extent arises from the vapour of certain hydrocarbons by which the diluted modern gas has been, as the saying is, "enriched," or brought up to such a standard as will pass, and these vapours are apt to condense long before they arrive at the consumer. Hence the statement recently made by the Chairman of the London County Council that "tests made with the portable photometer, although unrecognised by law, continue to show that a large amount of the gas supplied at other than the testing stations is considerably below the statutory requirements of 16 candles," and the further statement of the chemist to the County Council that "the effect of the present system of enrichment is partial and confined to the proximity of the testing places." We not only get the poison, but we fail to get the light.

